# Supplementary material for: A stochastic daily weather generator for perennial crop simulations in tropical Malaysia
Source: PLoS One. 2026 Feb 13;21(2):e0338833. doi: 10.1371/journal.pone.0338833 (PMC12904454; doi:10.1371/journal.pone.0338833)
Supplement: S1 File — (PDF) [file pone.0338833.s001.pdf]

S1. Supplementary Methods

S1.1 Air temperature generation

Autoregressive temperature modeling

Daily air temperatures exhibit temporal persistence, with each day's temperature strongly depending on the previous day's temperature. To capture this temporal dependence, a first-order autoregressive AR(1) process is employed to represent the day-to-day autocorrelation in temperature variability. Higher-order AR models can be used, but studies have demonstrated that they offer only marginal improvements in accuracy [1,2]. Additionally, these higher-order models come at the cost of increased model complexity due to the inclusion of more lagged variables, thereby making them more difficult to use. Moreover, the AR(1) process has been extensively validated for temperature simulations under diverse climatic conditions [3,4].

Temperature (T) generation for both minimum (Tmin) and maximum (Tmax) air temperatures for each month m requires four parameters: mean ($\mu_{T,m}$), standard deviation ($\sigma_{T,m}$), lag-1 autocorrelation ($\rho_{T,m}$), and skewness ($\gamma_{T,m}$). For each month m, the AR(1) process structures the temperature sequence as

$$T_{\mathrm{t}} = c_{T,m} + \rho_{T,m} T_{\mathrm{t}-1} + \epsilon_{T,t}^{(m)} \tag{S1.1}$$

where T_t and T_{t-1} are temperatures on day t and the previous day t-1, respectively; $c_{T,m}$ is the constant term, determined as $c_{T,m} = \mu_{T,m}(1 - \rho_{T,m})$; and $\epsilon_{T,t}^{(m)}$ is the error (residuals) term. To account for autocorrelation effects, the standard deviation of $\epsilon_{T,t}^{(m)}$, denoted by $\sigma_{\epsilon,T,m}$, is determined as $\sigma_{\epsilon,T,m} = \sqrt{\sigma_{T,m}^2 \left(1 - \rho_{T,m}^2\right)}$ [5].

Skew Normal (SN) distribution for residuals

The error (residual) term $\epsilon_{T,t}^{(m)}$ in Eq. S1.1 is sampled from an SN distribution [6,7]. Notably, $\epsilon_{T,t}^{(m)}$ is generated daily but drawn from a month-specific distribution. Although each day *t* possesses a unique residual value, the distribution parameters (*e.g.*, skewness $\gamma_{T,m}$, standard deviation $\sigma_{T,m}$, etc.) remain constant within each calendar month m. This approach allows the residuals to capture daily stochastic variability while preserving the underlying monthly statistical structure.

The probability density function (pdf) of the SN distribution is expressed as

$$f_T(\epsilon) = (2/\omega_m)\phi[(\epsilon - \xi_m)/\omega_m]\Phi[\alpha_m(\epsilon - \xi_m)/\omega_m] \quad \text{(S1.2)}$$

where ξ_m, ω_m, and α_m are the location, scale, and shape parameters, respectively; and $\varphi(\cdot)$ and $\Phi(\cdot)$ represent the standard normal pdf and cumulative distribution function (cdf), respectively. These parameters were estimated from the monthly target moments using the method of moments. Specifically, the shape parameter $\alpha_m = \delta_m/\sqrt{1-\delta_m^2}$, where $\delta_m = \text{sgn}(\gamma_{T,m})\sqrt{0.5\pi \cdot n_1/n_2}$, with $n_1 = |\gamma_{T,m}|^{2/3}$ and $n_2 = n_1 + [(4-\pi)/2]^{2/3}$. The notation sgn(·) represents the sign function, ensuring δ_m maintains the same sign as $\gamma_{T,m}$. The scale (ω_m) and location (ξ_m) parameters are calculated as $\omega_m = \sigma_{T,m}/\sqrt{(1-2\delta_m^2/\pi)}$ and $\xi_m = \mu_{T,m} - \omega_m\delta_m\sqrt{2/\pi}$.

However, the SN distribution becomes numerically unstable when $|\gamma_{T,m}|$ exceeds 0.995272 [7]. In such conditions, $\epsilon_{T,t}^{(m)}$ is instead sampled from an F-distribution, which is suited for modeling extreme right skewness [8]. The F-distribution is parameterized by two degrees of freedom: the numerator (df_1) and denominator (df_2). In this study, df_2 was fixed at 500, a choice motivated by both theoretical and computational considerations. A large value of df_2 ensures that the distribution tail decays smoothly, thereby preventing numerical overflow, while maintaining flexibility in the skewness representation. Johnson et al. [9] demonstrated that sufficiently large df_2 values minimize the denominator's influence, making skewness primarily dependent on the numerator df_1. This relationship enables the closed-form computation of df_1. Empirical testing further supports this choice, as df_2 values significantly smaller

than 500 (*e.g.*, 100) can lead to erratic tail behavior, whereas larger values (*e.g.*, 1000) provide diminishing or negligible gains in terms of numerical stability. For left-skewed distributions ($\gamma_{T,m} < 0$), residuals are generated by negating samples from the F-distribution fitted to $|\gamma_{T,m}|$. This reflection method, which is commonly used for handling asymmetric distributions, preserves the skewness magnitude while reversing its direction [7].

S1.2. Wind speed generation

Autoregressive wind speed modeling

Similar to air temperature, the daily wind speed (X) data also exhibit a serial correlation. An AR(1) model is used to capture this temporal dependence. For each calendar month m, the model requires three key wind speed parameters: mean ($\mu_{X,m}$), standard deviation ($\sigma_{X,m}$), and lag-1 autocorrelation ($\rho_{X,m}$). The wind speed on day t (X_t) is generated using an AR(1) model as

$$X_{\mathrm{t}} = c_{X,m} + \rho_{X,m} X_{\mathrm{t}-1} + \epsilon_{X,t}^{(m)} \tag{S1.3}$$

where $c_{X,m} = \mu_{X,m}\left(1 - \rho_{X,m}\right)$ is a constant term, and $\epsilon_{X,t}^{(m)}$ is the daily error (residuals) term. Consistent with the air temperature modeling approach, the wind speed residuals vary daily but are sampled from a month-specific Weibull distribution with fixed parameters for month m. The standard deviation of these residuals, $\sigma_{\epsilon,X,m}$, is adjusted for autocorrelation effects as $\sigma_{\epsilon,X,m} = \sqrt{\sigma_{X,m}^2 \left(1 - \rho_{X,m}^2\right)}$.

Weibull distribution for residuals

The wind speed residuals $\epsilon_{X,t}^{(m)}$ in Eq. S1.3 are sampled from a Weibull distribution, which has been estensively used in wind speed modeling applications [10–12]. The Weibull distribution is characterized by the following pdf:

$$f_X(\epsilon) = (k_m/\lambda_m)(\epsilon/\lambda_m)^{k_m - 1} \exp[-(\epsilon/\lambda_m)^{k_m}] \tag{S1.4}$$

where k_m and λ_m are the shape and scale parameters, respectively. The shape parameter k_m is calculated using an empirically derived relationship proposed by Justus et al. [13]: $k_m = \left(\sigma_{\epsilon,X,m}/\mu_{X,m}\right)^{-1.086}$, and the scale parameter is computed as $\lambda_m = \mu_{X,m}/\Gamma\left(1 + 1/k_m\right)$, where $\Gamma(\cdot)$ represents the gamma function.

The AR(1) model (Eq. S1.3) requires residuals with a zero mean, but the Weibull distribution inherently produces only positive values. Consequently, the residuals are generated through a two-step location-shift transformation: 1) values are first drawn from the Weibull distribution with the fitted parameters k_m and λ_m, then 2) the theoretical mean of the Weibull distribution ($\mu_{Weibull} = \lambda_m \times \Gamma(1 + 1/k_m) = \mu_{X,m}$) is subtracted from each sampled value to produce zero-mean residuals. This transformation creates residuals that follow a location-shifted Weibull distribution by maintaining the shape parameter (k_m) and variance while shifting only the location to achieve the required zero mean. This preserves the relative dispersion and skewness properties of wind speed variability, so that the resulting residuals encompass both positive and negative values symmetrically distributed around zero, thus ensuring compatibility with the AR(1) model.

S1.3. Rainfall generation

Rainfall generation follows a two-stage process. In the first stage, the model generates rainfall amounts for wet days, after which, in the second stage, the temporal distribution of rainfall amounts is determined based on a two-stage Markov chain process.

Stage 1. Modeling rainfall depths

Daily rainfall amounts for wet days follow a Gamma distribution defined by its cdf as

$$F_{Gam,R}(r_m) = \gamma(s_m, r_m/\theta_m)/\Gamma(s_m) \tag{S1.5}$$

where r_m is the monthly rainfall amount; s_m and θ_m are the shape and scale parameters for month m, respectively; $\Gamma(\cdot)$ is the Gamma function; and $\gamma(\cdot,\cdot)$ is the lower incomplete gamma function. The shape

parameter s_m governs the skewness and dispersion of rainfall amounts. To characterize its variability, Gamma distributions were first fitted to observed wet-day rainfall data for each calibration site, year, and calendar month, yielding a large set of empirical s_m estimates.

Analysis of these fitted shape parameter estimates showed an asymmetric, right-skewed distribution, with three-quarters of the data concentrated between 0.6 and 1.0. To capture this variability, a Generalized Extreme Value (GEV) distribution was fitted to the shape parameter estimates. The GEV is particularly suitable for modeling heavy-tailed behavior and extremes often found in hydrological data. The cdf of the GEV is given by

$$F_{GEV,R}(x) = \exp\{-[1 + c\,(x-a)/b]^{-1/c}\} \quad \text{(S1.6)}$$

for $c \neq 0$, where a, b, and c are the location, scale, and shape parameters, respectively. Fitting the GEV distribution to the Gamma shape values yielded the following parameter estimates: a=0.50, b=0.17, and c=0.14.

With the GEV parameters established, daily rainfall amounts are generated through the following steps. First, a Gamma shape parameter is obtained by drawing a random number from a uniform distribution [0, 1] and transforming it using the inverse cdf of the GEV distribution with parameters a=0.50, b=0.17, and c=0.14. If the drawn shape value is negative, it is rejected, and a new value is sampled. Next, the corresponding Gamma scale parameter is calculated as $\theta_m = \mu_{R,m}/s_m$, where $\mu_{R,m}$ is the observed mean wet-day rainfall for month m. Finally, with both Gamma parameters established, the rainfall amount for day t in month m ($R_{t,m}$) is generated by applying the inverse cdf of the Gamma distribution to another random number drawn from a uniform distribution [0, 1].

Stage 2. Distribution of wet and dry days

The temporal sequencing of wet (W) and dry (D) days follows a first-order two-state Markov chain process, where the probability of rainfall on a given day depends only on whether the previous day was wet or dry. This stochastic process is characterized by two key transition probabilities: P_{WW}, representing the probability (P) that a wet day follows another wet day (persistence of wet conditions),

and P_{WD}, representing the probability that a wet day follows a dry day (transition from dry to wet conditions). These transition probabilities capture the temporal dependence inherent in rainfall patterns, reflecting both the clustering of wet days and the likelihood of transitions between wet and dry states.

The simulation procedure operates sequentially as follows. For each day in the time series, the model first identifies the precipitation state of the previous day. Based on this information, the appropriate transition probability is selected: P_{WW} if the previous day was wet, or P_{WD} if the previous day was dry. A random number u is then drawn from a uniform distribution [0, 1]. If u is less than the selected transition probability, the current day is classified as wet; otherwise, it is classified as dry.

When a day is determined to be wet through this process, the model assigns a specific rainfall amount by sampling from the previously generated distribution of daily rainfall values. This sequential procedure continues for each day in the simulation period, producing a synthetic rainfall time series that preserves both the statistical characteristics of wet-dry day transitions and the distributional properties of rainfall amounts observed in the historical record.

S1.4. Distributions

Table 1 summarizes MisaGen's weather generation methodology, showing for each meteorological variable the temporal modeling structure, probability distributions used, monthly parameters that users must provide or calculate, and the methods for parameter estimation.

Table 1. Summary of MsiaGen weather generation methodology, showing temporal modeling approaches, statistical distributions, required parameters, and estimation methods for each meteorological variable.

Weather varable	Temporal Structure	Distribution(s)	Required Monthly Parameters[2]	Parameter Estimation	Key Features
Air Temperature (Tmin, Tmax)	First-order autoregressive AR(1) process	Skew Normal for residuals[2]	Mean ($\mu_{T,m}$), standard deviation ($\sigma_{T,m}$), lag-1 autocorrelation ($\rho_{T,m}$), skewness ($\gamma_{T,m}$)	Method of moments	Captures day-to-day persistence. Handles observed asymmetry in tropical temperatures.
Wind Speed	First-order autoregressive AR(1) process	Weibull for residuals[3]	Mean ($\mu_{X,m}$), standard deviation ($\sigma_{X,m}$), lag-1 autocorrelation ($\rho_{X,m}$)	Method of moments with empirical relationship by Justus et al. [13]	Captures temporal dependence. Location-shift transformation produces zero-mean residuals.
Rainfall	Two-stage process:<br>1. Rainfall amount generation<br>2. Wet/dry day sequencing	Stage 1: Gamma[4]<br>Stage 2: First-order two-state Markov chain	Stage 1: Rainfall (r_m)<br>Stage 2: Transition probabilities (P_{WW}, P_{WD})[5]	Gamma shape parameters (s_m) drawn from calibrated GEV (location=0.50, scale=0.17, and shape=0.14).<br>Gamma scale parameter (θ_m) computed as mean wet-day rainfall ($\mu_{R,m}$) / s_m.	Variable shape parameter introduces realistic stochastic variability. Markov chain captures wet/dry persistence with minimal parameters.

[1] All parameters are required for each calendar month (m=1 to 12). Users can extract these from local weather data or specify custom values for scenario analysis.
[2] F-distribution used when $|\gamma_{T,m}| > 0.995272$ to maintain numerical stability.
[3] Weibull residuals are location-shifted to achieve zero mean required for AR(1) process.
[4] Gamma distribution characterizes wet-day rainfall amounts; shape parameter treated as random variable.
[5] P_{WW} = probability wet day follows wet day; P_{WD} = probability wet day follows dry day.

References

1. Wilks DS. Multisite downscaling of daily precipitation with a stochastic weather generator. Clim Res. 1999;11: 125–136. doi:10.3354/cr011125

2. Mearns LO, Rosenzweig C, Goldberg R. Mean and variance change in climate scenarios: Methods, agricultural applications, and measures of uncertainty. Clim Change. 1997;35: 367–396. doi:10.1023/A:1005358130291

3. Richardson CW. Stochastic simulation of daily precipitation, temperature, and solar radiation. Water Resour Res. 1981;17: 182–190. doi:10.1029/WR017i001p00182

4. Wilks DS, Wilby RL. The weather generation game: A review of stochastic weather models. Prog Phys Geogr. 1999;23: 329–357. doi:10.1177/030913339902300302

5. Box GEP, Jenkins GM. Time series analysis: Forecasting and control. Holden-Day; 1976.

6. Azzalini A. A class of distributions which includes the normal ones. Scand J Stat. 1985;12: 171–178.

7. Azzalini A. The Skew-Normal and Related Families. Cambridge University Press; 2013.

8. Wilks DS. Statistical methods in the atmospheric sciences. 3rd ed. Academic Press; 2011.

9. Johnson NL, Kotz S, Balakrishnan N. Continuous univariate distributions. 2nd ed. Wiley; 1995.

10. Justus CG, Hargraves WR, Yalcin A. Nationwide assessment of potential output from wind-powered generators. J Appl Meteorol. 1976;15: 673–678. doi:10.1175/1520-0450(1976)015%3C0673:NAOPOF%3E2.0.CO;2

11. Seguro JV, Lambert TW. Modern estimation of the parameters of the Weibull wind speed distribution for wind energy analysis. J Wind Eng Ind Aerodyn. 2000;85: 75–84. doi:10.1016/S0167-6105(99)00122-1

12. Aljeddani SM, Mohammea M. A novel approach to Weibull distribution for the assessment of wind energy speed. Alex Eng J. 2023. doi:10.1016/j.aej.2023.07.027

13. Justus CG, Hargraves WR, Mikhail A, Graber D. Methods for estimating wind speed frequency distributions. J Appl Meteorol. 1978;17: 350–353. doi:10.1175/1520-0450(1978)017%3C0350:MFEWSF%3E2.0.CO;2
