# Supplementary material for: A stochastic daily weather generator for perennial crop simulations in tropical Malaysia
Source: PLoS One. 2026 Feb 13;21(2):e0338833. doi: 10.1371/journal.pone.0338833 (PMC12904454; doi:10.1371/journal.pone.0338833)
Supplement: S1 Table — Values represent percentage change in annual fresh fruit bunch (FFB) yield relative to baseline simulations using unmodified observed wind speeds. YAP = years after planting. Negative values indicate yield declines; positive values indicate yield increases. (PDF) [file pone.0338833.s002.pdf]

Table 2. Simulated oil palm yield responses to ±20% daily wind speed manipulations at Kerayong (a) and Kalumpong (b) across three planting densities. Values represent percentage change in annual fresh fruit bunch (FFB) yield relative to baseline simulations using unmodified observed wind speeds. YAP = years after planting. Negative values indicate yield declines; positive values indicate yield increases.

a) Kerayong

	136 palms ha^{-1}		160 palms ha^{-1}		185 palms ha^{-1}	
**YAP**	**-20%**	**+20%**	**-20%**	**+20%**	**-20%**	**+20%**
8	-1.03	-0.95	0.69	-0.83	1.78	0.37
9	-2.45	2.22	1.42	-1.71	-0.04	-1.32
10	-0.03	1.23	3.35	-2.94	1.80	-1.03
11	-0.77	-0.47	2.85	-4.25	2.86	-2.07
12	0.15	-0.25	6.70	-4.84	2.05	-2.70
13	1.35	-1.13	6.26	-1.18	2.29	-0.59
14	2.01	-0.86	3.46	-2.42	2.74	-2.57
15	-0.62	-1.12	7.55	-6.02	5.09	-2.93
16	2.80	-0.45	5.36	-5.16	3.91	-2.73
17	2.80	-2.22	6.52	-4.51	3.84	-1.86
18	0.14	-0.55	5.14	-3.63	3.59	-2.47
19	-1.36	-0.09	3.53	-2.45	1.49	-1.30
**Mean**	**0.25**	**-0.39**	**4.40**	**-3.33**	**2.62**	**-1.77**

b) Kalumpong

	136 palms ha^{-1}		160 palms ha^{-1}		185 palms ha^{-1}	
**YAP**	**-20%**	**+20%**	**-20%**	**+20%**	**-20%**	**+20%**
8	4.15	-7.34	4.53	-3.66	3.89	-2.66
9	13.10	-7.83	8.70	-7.64	8.24	-9.08
10	11.92	-8.71	12.44	-8.64	11.03	-5.85
11	3.58	-3.83	0.92	-6.35	2.28	-5.42
12	9.83	-5.08	12.22	-5.58	14.13	-4.53
13	5.21	-3.37	5.30	-3.65	4.66	-5.25
14	6.02	-4.91	7.07	-4.19	6.67	-4.19
15	7.51	-4.24	7.47	-4.39	6.82	-4.20
16	4.39	-6.53	4.60	-6.50	5.63	-6.32
17	13.31	-4.24	12.36	-7.69	12.54	-8.32
18	11.57	-8.65	11.00	-10.29	9.82	-12.15
19	8.86	-9.21	10.16	-8.74	12.00	-8.69
20	7.77	-5.39	7.61	-5.60	9.18	-4.84
21	14.20	-8.63	13.08	-10.68	14.33	-9.59
22	9.60	-9.76	10.45	-9.79	11.93	-9.41
**Mean**	8.28	6.16	8.07	6.44	8.14	6.39
