# Supplementary material for: A stochastic daily weather generator for perennial crop simulations in tropical Malaysia
Source: PLoS One. 2026 Feb 13;21(2):e0338833. doi: 10.1371/journal.pone.0338833 (PMC12904454; doi:10.1371/journal.pone.0338833)
Supplement: Attachment — Submitted filename: Response to reviewers.docx [file pone.0338833.s009.docx]

**PONE-D-25-41380**

**A stochastic daily weather generator for perennial crop simulations in tropical Malaysia**

**RESPONSE TO REVIEWERS**

**14 OCT 2025**

We are grateful to the reviewers for their suggestions and comments. We have addressed each point as follows:

**REVIEWER 1**

| **No.** | **Reviewer’s comments** | **Our response** |
| --- | --- | --- |
| 1. | Please, check the keywords: L. 39 there should be "distribution", and not use just abbreviation "MsiaGen". | Corrected “distribution” spelling error, and removed “MsiaGen” from the list of keywords (line 39). |
| 2. | In the chapter Study Sites, L. 87, please introduce there why just 23 stations are considered (it is later in L. 102-103.  Please, introduce there the climate zonation according to the Koppen classification, the potential evapotranspiration and a potential water budget. | Moved lines 102–103 (location based on original manucscript) to lines 87-92 (in the revised manuscript) to introduce earlier why the 23 sites were selected.  Added lines 107–115 about climate zonation and information about PET and potential water budget, with four new supporting references [13, 26-28]. |
| 3. | L. 220, please, introduce the definition of dry and wet days. | Defined wet days as days with rain ≥0.05 mm and dry days as those with rain <0.05 mm (line 162). |
| 4. | The model Msia Gen should be well described in Methodology, do not do it in Conclusions (L.767-770 are not necessary), in the chapter Conclusions go directly to your achievements. | Conclusion section, line 952: Removed the first two sentences (lines 767–770 in the original manuscript), as recommended, to avoid methodology repetition. |

**REVIEWER 2**

| **No.** | **Reviewer’s comments** | **Our response** |
| --- | --- | --- |
| 1. | Test an alternative rainfall distribution (e.g., Generalized Pareto, mixed-exponential) for comparison, even if briefly, to show MsiaGen’s trade-offs. | Added a new section titled, “Comparative evaluation of alternative distributions” under Discussion (lines 748–807) to discuss the comparative analysis between fitting Gamma and two suggested alternatives: Generalized Pareto (GPD) and Mixed Exponential (MixExp) distributions to the observed rainfall.  Results (new Table 2, showing RMSE values of distribution fits for each site) indicate the superiority of the simpler and more practical Gamma distribution over MixExp and GPD. Gamma was superior for 61% and MixExp 39% of the 23 sites. GPD fitted very poorly across all sites. Even at sites MixExp was superior over Gamma, the mean RMSE difference was modest. This is despite fitting MixExp (as well as GPD) using maximum likelihood estimation (MLE), which is a more accurate fitting method over the method of moments (MoM).  Lines 783–807, in particular, highlight the importance of fitting distributions using MoM in MsiaGen, and why Gamma is most suited for this purpose. Results from the oil palm yield simulation further fortify the justification of using Gamma distribution. |
| 2. | Add explicit metrics for wet/dry spell length reproduction and compare to observations. | We have added comprehensive quantitative validation of wet/dry spell length distributions (Supplementary Results Figs. S2.4–S2.5).  At the aggregate level (all sites pooled), box plots and empirical cumulative distribution functions (ECDF) demonstrate close alignment between observed and generated spell length distributions when pooled across all sites (Supplementary Results Fig. S2.4).  Site-level analysis (per site) shows mean wet spell lengths of observed versus generated, and mean dry spell lengths of observed versus generated across 23 sites (Supplementary Results Fig. S2.5.  We have added discussion of these findings in lines 632–655, which reveals that while the model adequately captures typical spell length distributions, systematic biases exist at sites with extreme persistence characteristics.  However, while slight overestimation occurs at sites with longer dry spells, the successful yield simulations (Fig. 11) demonstrate that this bias does not compromise the model’s representation of water availability patterns relevant for oil palm productivity.  We further added lines 337–342 and a new Fig. 4 to determine how closely generated rainfall transition probabilities (PWW and PWD) matched observations. Visual inspection of model agreement and goodness-of-fit metrics (NMAE, NMBE, and KGE) showed that generated rainfall transition probabilities for wet-wet and wet-dry periods closely matched observed values, with small model error and bias and high agreement scores. Model agreements between generated and observed data were also plotted (Fig. 4). |
| 3. | If possible, include one or two sites with ≥20 years of data to test parameter stability. | Our study already included two independent sites with long-term records (Kerayong with 22 years and Kalumpong with 23 years, lines 253 and 261) that were specifically excluded from calibration/validation datasets. Both these sites were used in oil palm yield simulations (lines 251–254), and comparison results between generated weather over two decades and observed weather are presented in Figs. 9–10.  The use of both sites demonstrate an important methodological distinction: the parameters demonstrated temporal stability (producing consistent statistical properties when applied over 22-23 years; Figs. 9-10), but they still exhibit the systematic underestimation of extremes (lines 384–390, 485–487, 516–519).  This occurs likely because the parameters were derived from the short-record (3–5 year) calibration sites that lacked sufficient sampling of rare extreme events. Thus, the limitation is not parameter instability over time, but rather incomplete characterization of distribution tails due to short calibration records.  To clarify this important distinction, we have revised relevant sections as indicated below:   1. Methods section (lines 270–273): Added:  “The long-term weather records generated for Kerayong and Kalumpong (22–23 years) provide an additional benefit. They allow for an assessment of the temporal stability of the weather generator model parameters. This is possible because these long-term weather series were generated using parameter values that were originally calibrated on sites with only 3–5 years of data.” 2. Results section (lines 516–519): Added:  “Generated weather at Kerayong and Kalumpong over 20 year periods (Figs. 9–10) produced trends similar to those observed at calibration and validation sites: good agreement for common conditions but underestimation of extreme rainfall (>80–100 mm) and wind speeds (>3–4 m s–1) (compare Fig. 9e–h with Fig. 5c–d).” 3. Discussion section (lines 610–621): Revised paragraphs:  “Calibration parameters derived from these 3–5 years records, however, successfully generated weather for the 22–23 year periods at Kerayong and Kalumpong (Figs. 9–10). They produced trends similar to those observed at calibration and validation sites (Fig. 5, 7, and Supplementary Results Figs. S2.1–S2.3). The results demonstrate that the parameters, calibrated using short-term records, exhibit sufficient temporal stability to be reliably applied in long-term agricultural modeling and scenario analysis.  “However, having temporal stability does not resolve the issue of inadequate extreme event sampling. The short calibration period provided insufficient observations of rare events (e.g., only 2% of days exceeded 60 mm rainfall; Fig. 8a) that are needed to accurately characterize distribution tails. This likely led to underestimation of extremes, but despite this limitation, the model remains suitable for oil palm applications where cumulative seasonal patterns, which MsiaGen accurately captures, drive yield responses more than rare extreme events (Fig. 11).” 4. Discussion section (lines 869–871): Added:  “Weather generation for over 20 years period at both Kerayong and Kalumpong plantation sites using parameters derived from short-term records confirms temporal stability of the parameters.” 5. Conclusion (lines 962–964): Added:  “This long-term application confirmed temporal stability of model parameters while also demonstrating that the systematic underestimation of extremes (likely attributable to short calibration periods) persists consistently across decades.” |
| 4. | Provide more detail on how inaccuracies in extremes (e.g., rainfall >100 mm) might impact oil palm yield modeling outcomes. | 1. Added new sub-section “Rainfall sensitivity analyses” under Methods section (lines 274–297) to assess oil palm yield simulations sensitity to three sets of scenario. By manipulating rainfall through removal and redistribution between intensity classes, these scenarios quantified yield responses to increasing rainfall losses and the relative importance of rainfall intensity distribution for oil palm productivity. 2. Added new sub-section “Rainfall intensity distribution effects” under Results section (lines 536–566). At the end, the results indicated that maintaining total annual rainfall is more critical than preserving specific intensity distributions. However, oil palm yields showed greater sensitivity to reductions in lower-intensity rainfall than to equivalent reductions in higher-intensity rainfall, indicating that frequent, moderate rainfall events are more important for sustaining productivity than infrequent extreme events. 3. Added new Fig. 12 showing the effects of various rainfall scenarios on oil palm yield. 4. Added discussion on the rainfall sensitivity analyses (Discussion section, lines 898–930). 5. In addition to rainfall extremes, we also tested wind speed sensitivity analysis of oil palm yields (lines 691–717) and added Supplementary Results Table S2.1.   The various rainfall scenarios demonstrated that oil palm yields depend primarily on total water availability (deficit scenarios reduced yields 5-45%) but also on intensity distribution. Redistributing rainfall from higher- to lower-intensity events increased yields 1-8% despite unchanged totals, reflecting oil palm’s greater sensitivity to frequent, moderate rainfall (95% of rainy days, 75-78% of annual total) than to rare extremes that generate runoff. Thus, MsiaGen’s underestimation of extreme events does not compromise yield predictions, as the model accurately captures the frequent, lower-intensity rainfall that dominates productivity.  Additionally, the model’s underestimation of extreme wind speeds has small practical impact on oil palm yields for reasons discussed in lines 691–717. |
| 5. | Move highly technical derivations (e.g., parameter transformations for Skew Normal) to appendices/supplementary material. This keeps the main text accessible. | Moved the technical details about the equations and parameter derivations of the various distributions to the Supplementary Methods S1.1–S1.3. In their place in the main text, only key methodology steps/details are briefly discussed (lines 120–189).  Added a new table to summarize key setps and user input requirements (Supplementary Methods Table S1.1). |
| 6. | Typo in keywords (“distibution” → “distribution”), improve figure captions (add sample sizes, error bars). | 1. Corrected the misspelling for “distribution” (line 39). 2. Added sample sizes to Figs. 2–10 (including all figures in the Supplementary Results S2). 3. We appreciate the reviewer’s suggestion to include uncertainty estimates. We have considered whether our figures would benefit from having error bars:  - Figs. 2, 5–10: These present distributional characteristics (box plots, QQ plots, ECDF plots) or summary statistics across sites where the current visualization formats (box plots, density distributions) already convey variability more comprehensively than error bars would. Moreover, the QQ and ECDF plots already show full distributional comparisons where error bars are not applicable to these plot types. - Error bars for Fig. 3 is not applicable as it is meant to show the inverse linear relationship between max. air temperature and rainfall. - Fig. 4 (new) displays monthly rainfall transition probabilities (P_WW_ and P_WD_), where each point represents a probability estimate calculated by pooling all relevant transition events for that site-month combination across the 3-5 year record period. These are statistical estimates from finite samples rather than replicated measurements, and thus do not have error bars. - Fig. 11 (yield simulations): While we have replicate data for the observed oil palm yields, the primary purpose of Fig. 11 is to validate agreement between oil palm yield simulations using generated vs. observed weather (red vs. blue lines), rather than to assess their accuracy in matching observed yields (*i.e.*, assess crop model accuracy).  Adding error bars to yield observations would shift focus from this key comparison—whether generated weather produces equivalent simulation outputs to observed weather—to crop model performance, which was validated elsewhere in our previous work (Teh et al., 2024; [43]). We believe the current presentation best highlights the intended validation purpose. - Fig. 12 (new) (rainfall scenarios): These show deterministic model responses to specific rainfall manipulations, where each scenario produces a single yield outcome; thus, error bars are not possible. |
| 7. | The calibration is based on relatively short weather records (3–5 years per site). This limitation likely contributes to underperformance in simulating extremes. It may be justified more clearly why this truncation was necessary and, if possible, provide a sensitivity analysis or at least include one or two sites with longer datasets to test parameter stability | We explained in lines 89–92 in “Study sites” sub-section under methodology section: “…to prevent bias from uneven data lengths, all records were standardized to a 3–5 year period (1095–1827 days). This ensured balanced representation across regions, avoiding disproportionate influence from stations with longer historical data.”  We additionally included two sites (Kerayong and Kalumpong) with long-term weather data of 22 and 23 years, respectively, in our paper for oil palm yield simulations, including temporal parameter stability (see point 3 above). |
| 8. | The manuscript acknowledges underestimation of high rainfall (>100 mm) and wind speeds (>3–4 m s⁻¹). Since these extremes are critical for agriculture, especially for perennial crops like oil palm, the authors should:  • Provide explicit quantitative evaluation of how frequently such events are misrepresented.  • Explore or at least compare with alternative distributions (e.g., Generalized Pareto, mixed-exponential) that may better capture heavy tails. | We like to point out that our results indicate that oil palm yields depend primarily on total water availability and on frequent, light and moderate rainfall rather than to rare rainfall extremes (see point 4 above).  Explicit quantitative evaluation of how frequently such events are misrepresented are presented in “Performance analysis across rainfall amount classes” sub-section under Results section (lines 450–477). In addition, Fig. 8 show the error for each rainfall class.  See point 2 above about accuracy assessment of wet/dry spell lengths reproduction, as well as wind speed extremes  See point 1 above about the inclusion of the comparative analysis using Gamma versus two other alterrnative (Generalized Pareto and Mixed-exponential) distributions. |
| 9. | The first-order Markov chain may inadequately represent monsoonal persistence. The discussion notes this but does not present quantitative validation. | Done as suggested. Please see point 2 above. |
| 10. | Spell length statistics (mean wet/dry spell length, frequency distribution) comparing observed vs. simulated data may be included. This would greatly strengthen confidence in MsiaGen’s ability to reproduce tropical rainfall characteristics. | Done as suggested. Please see point 2 above. |
| 11. | While the model is designed for Malaysia, it would be valuable to discuss explicitly whether MsiaGen can be extended to other equatorial regions with similar Af climates (e.g., Indonesia, Papua New Guinea). Even if not tested, a short discussion would help readers assess applicability. | Added lines 943–951 to discuss about the application of MsiaGen to other tropical regions. |
| 12. | The yield simulations show close agreement overall, but the manuscript does not quantify how misrepresentation of extremes propagates into yield estimates. The following would need clarification:  • Do errors in extreme rainfall or wind events materially affect modelled yields?  • Could yield risk assessments underestimate extreme-event-driven yield loss? | Done as suggested. Please see point 4 above on rainfall and wind speed sensitivity analysis.  Please also note that we wrote that MsiaGen weather generator is primarly intended for crop simulations, rather than for predicting extreme events such as in flood risk assessment (lines 745–747, 940–942). |
| 13. | Consider moving some of these derivations to supplementary material and instead provide a concise summary table of distributions, parameters, and rationales in the main text. Figures showing ECDFs for every site are informative but numerous. Suggest moving some panels to Supplementary Information and retaining representative sites in the main text. | Done as suggested. Please see point 5 above.  A table is provided, as suggested, to summarize MisaGen’s weather generation methodology, showing for each meteorological variable the temporal modeling structure, probability distributions used, monthly parameters that users must provide or calculate, and the methods for parameter estimation (Supplementary Methods Table S1.1). |
| 14. | The manuscript states that full datasets are available but relies heavily on short station records. It may be clarified whether raw station data are archived in a national database and if readers can access them directly, or whether only processed datasets are provided. | We provided the source of the weather data used in the study (lines 92–94). |
