# Supplementary material for: A stochastic daily weather generator for perennial crop simulations in tropical Malaysia
Source: PLoS One. 2026 Feb 13;21(2):e0338833. doi: 10.1371/journal.pone.0338833 (PMC12904454; doi:10.1371/journal.pone.0338833)
Supplement: Attachment — Submitted filename: Response 2 to reviewers.docx [file pone.0338833.s010.docx]

**PONE-D-25-41380R1**

**A stochastic daily weather generator for perennial crop simulations in tropical Malaysia**

**RESPONSE TO REVIEWERS (2ND REVISION)**

**7 NOV 2025**

We are grateful to the reviewers for their suggestions and comments. We have addressed each point as follows:

**REVIEWER 1**

| **No.** | **Reviewer’s comments** | **Our response** |
| --- | --- | --- |
| 1. | Please, keep the limit of 300 words in the abstract. | Lines 17-38. Abstract shortened to 298 words. |
| 2. | In keywords, I think, it is better to use "oil palm yield" | Line 39. Changed keyword “oil palm” to “oil palm yield”. |
| 3. | Considering the structure of your paper, probably, better seems to use the standard structure required by the journal guidelines: Material and methods .... | Line 84. Section title “Methodology” changed to “Materials and Methods”, following journal guidelines. |
| 4. | I prefer the chapter "Study site" (instead Study sites), descripting the area of your interest, those 23 meteorological stations are just parts of this study site. | Line 85. Changed section title to “Study site”, as suggested. |
| 5. | Please discuss the honogeinity of the analysed data-sets. | We appreciate the reviewer’s suggestion to assess homogeneity. Given that our records spanned only 3–5 years per site, we did not conduct formal homogeneity tests because the study objective was to develop and validate a stochastic daily weather generator (MsiaGen) for use in agricultural modelling, not to infer climatic trends. The generator only needs internally consistent short-term weather statistics (mean, variance, skewness, autocorrelation) for calibration, not long-term homogeneity.  We assumed within-month stationarity at each site and estimated parameters independently (no pooling across stations). This approach confines parameter estimation to local data, thereby preventing biases that could arise from combining climatically diverse sites and thus reducing sensitivity to spatial inhomogeneity.  We now state this assumption and rationale in the Materials and Methods section (lines 121–128), and we note the limitation in the Discussion section (lines 627–635).  The consistent calibration and validation results, as well as the multi‑decadal oil palm simulations, support that the monthly statistics are representative for our intended application. |
| 6. | And, discuss also when the model performance has comparable value in all the climate district of your study. | The comparability of model performance across sites is documented throughout in the Results and Discussion sections.  In brief, performance is highly comparable at the monthly scale across all 23 sites, with low errors (NMAE <1.2% for Tmin/Tmax, <2.4% for wind speed, <1.8% for rainfall), negligible bias, and strong agreement (KGE ≥0.95 for rainfall; ≥0.80 for temperatures; ≥0.70 for wind speed) for both calibration and validation sets (Fig. 2).  At the daily scale, agreement is comparably strong for the conditions that constitute >90% of observations: Tmin 21–28°C and Tmax 25–39°C, wind speeds up to about 3 m s^-1^, and rainfall below ~80 mm day^-1^, while divergence increases in the upper tails, notably for rainfall above 80–100 mm day^-1^ and wind speeds above 3–4 m s^-1^ (Figs. 5–6, 8).  These differences reflect tail‑model limitations, the short 3–5 year calibration windows, first‑order wet/dry sequencing, and climatological contrasts between calibration and validation sites.  Because the network spans mainly Köppen Af with Am enclaves rather than a single homogeneous ‘climate district,’ MsiaGen is parameterized per site. Comparability therefore refers to consistency of performance across this range of local climates.  For the intended perennial‑crop applications, the demonstrated comparability at monthly to seasonal scales is the critical criterion, as confirmed by multi‑decadal oil palm yield simulations at two independent sites that replicate observed temporal dynamics across planting densities (Fig. 11).  We added an explicit synthesis paragraph in the Discussion to make these points clear (lines 579–586). |
| 7. | The statement "MsiaGen is written in Julia programming language, and its full source code is available at: github.com/cbsteh/MsiaGen" belongs not to the Conclusions, it should be introduced in the chapter describing the model. | This statement now appears earlier in the “Materials and Methods” section in lines 134-135 (para 1, subsection “MsiaGen weather generation framework”). |

**REVIEWER 2**

No suggestions or corrections given by 2nd reviewer after first revision.
